# Supplementary material for: Sorting at embryonic boundaries requires high heterotypic interfacial tension
Source: Nat Commun. 2017 Jul 31;8:157. doi: 10.1038/s41467-017-00146-x (PMC5537356; doi:10.1038/s41467-017-00146-x)
Supplement: Supplementary file 2 — Supplementary Software 1 [file 41467_2017_146_MOESM2_ESM.zip › PottsModel/SrcPottsModel/doc/model/LatticeUtilities.html]

LatticeUtilities


JavaScript is disabled on your browser.


Skip navigation links


- Overview
- Package
- Class
- Use
- Tree
- Deprecated
- Index
- Help

- Prev Class
- Next Class

- Frames
- No Frames

- All Classes

- Summary:
- Nested |
- Field |
- Constr |
- Method

- Detail:
- Field |
- Constr |
- Method


model

## Class LatticeUtilities

- java.lang.Object
- - model.LatticeUtilities

- ---

    

  ```
  public class LatticeUtilities
  extends java.lang.Object
  ```

- - ### Constructor Summary

    Constructors

    Constructor and Description

    `LatticeUtilities()`
  - ### Method Summary

    All Methods Static Methods Concrete Methods

    | Modifier and Type | Method and Description |
    | --- | --- |
    | `static int[][][]` | `boundaryLattice(int width, int height, int cellSize)` |
    | `static int[][][]` | `boundaryLattice(int width, int height, int cellSize, int mediumSize)` |
    | `static int[][][]` | `checkeredLattice(int width, int height, int cellSize)` |
    | `static int[][][]` | `checkeredLattice(int width, int height, int cellSize, int mediumSize)` |
    | `static Cell.CellType` | `inverse(Cell.CellType c)` |
    | `static int[][][]` | `inverse(int[][][] lattice)` |
    | `static int[][][]` | `lattice(Lattice.Type t, int width, int height, int cellWidth, int cellHeight, int mediumWidth, int mediumHeight)` Returns lattice of cell IDs and Types of dimension (2 \* width \* height) where lattice[0][i][j] returns the cell ID of pixel at position (i,j) and lattice[1][i][j] returns the cell type of pixel at position (i,j) |
    | `static void` | `main(java.lang.String[] args)` |
    | `static int[][][]` | `makeRound(int[][][] pLattice, int radius)` |
    | `static int[][][]` | `readLattice(java.lang.String path)` |
    | `static void` | `writeLattice(Lattice l, java.lang.String path)` |

    - ### Methods inherited from class java.lang.Object

      `equals, getClass, hashCode, notify, notifyAll, toString, wait, wait, wait`

- - ### Constructor Detail


    - #### LatticeUtilities

      ```
      public LatticeUtilities()
      ```
  - ### Method Detail


    - #### checkeredLattice

      ```
      public static int[][][] checkeredLattice(int width,
                                               int height,
                                               int cellSize,
                                               int mediumSize)
      ```


    - #### boundaryLattice

      ```
      public static int[][][] boundaryLattice(int width,
                                              int height,
                                              int cellSize,
                                              int mediumSize)
      ```


    - #### checkeredLattice

      ```
      public static int[][][] checkeredLattice(int width,
                                               int height,
                                               int cellSize)
      ```


    - #### boundaryLattice

      ```
      public static int[][][] boundaryLattice(int width,
                                              int height,
                                              int cellSize)
      ```


    - #### inverse

      ```
      public static int[][][] inverse(int[][][] lattice)
      ```


    - #### inverse

      ```
      public static Cell.CellType inverse(Cell.CellType c)
      ```


    - #### lattice

      ```
      public static int[][][] lattice(Lattice.Type t,
                                      int width,
                                      int height,
                                      int cellWidth,
                                      int cellHeight,
                                      int mediumWidth,
                                      int mediumHeight)
      ```

      Returns lattice of cell IDs and Types of dimension (2 \* width \* height)
      where lattice[0][i][j] returns the cell ID of pixel at position (i,j) and
      lattice[1][i][j] returns the cell type of pixel at position (i,j)

      Parameters:
      :   `width` -
      :   `height` -
      :   `cellSize` -
      :   `mediumSize` - margin of medium

      Returns:


    - #### writeLattice

      ```
      public static void writeLattice(Lattice l,
                                      java.lang.String path)
                               throws java.io.IOException
      ```

      Throws:
      :   `java.io.IOException`


    - #### readLattice

      ```
      public static int[][][] readLattice(java.lang.String path)
                                   throws java.io.IOException
      ```

      Throws:
      :   `java.io.IOException`


    - #### makeRound

      ```
      public static int[][][] makeRound(int[][][] pLattice,
                                        int radius)
      ```


    - #### main

      ```
      public static void main(java.lang.String[] args)
      ```


Skip navigation links


- Overview
- Package
- Class
- Use
- Tree
- Deprecated
- Index
- Help

- Prev Class
- Next Class

- Frames
- No Frames

- All Classes

- Summary:
- Nested |
- Field |
- Constr |
- Method

- Detail:
- Field |
- Constr |
- Method
